# Long-term outcome of primary Papillary Urothelial Neoplasm of Low Malignant Potential (PUNLMP) including PUNLMP with inverted growth

**DOI:** 10.1186/s13000-015-0234-z

**Published:** 2015-03-13

**Authors:** Jay P Maxwell, Cheng Wang, Nicholas Wiebe, Asli Yilmaz, Kiril Trpkov

**Affiliations:** Department of Pathology and Laboratory Medicine, Calgary Laboratory Service and University of Calgary, Rockyview General Hospital, 7007 14 Street, Calgary, AB Canada

**Keywords:** PUNLMP, Recurrence, Progression, Low Grade Urothelial Carcinoma, Inverted Papilloma

## Abstract

**Background:**

Few larger studies have evaluated the long-term outcome after a diagnosis of papillary urothelial neoplasm of low malignant potential (PUNLMP), demonstrating a broad range of recurrence and progression rates. Additionally, no study has addressed the outcome of PUNLMP exhibiting inverted growth. We evaluated the long term clinical outcome of primary papillary urothelial neoplasm of low malignant potential (PUNLMP), including PUNLMP with inverted growth in a large single center study.

**Methods:**

We evaluated 189 primary PUNLMP (177 exophytic, 12 inverted), diagnosed from January 1, 2000 to December 31, 2009, in a centralized uropathology practice. We excluded PUNLMP diagnosed after a previous or with a concurrent urothelial neoplasm. Recurrence was defined as any subsequent urothelial neoplasm, regardless of the grade. Progression was defined as any subsequent higher-grade or invasive urothelial neoplasm. Recurrence and progression were established only if documented on a subsequent biopsy. Descriptive statistical analysis was performed using Microsof Excel software package.

**Results:**

The location of PUNLMP included bladder (187) and renal pelvis and ureter (1 each). After a median follow-up of 61 months (range, 9–128 months), 20.1% patients developed a recurrence. Recurrence with PUNLMP only was found in 9% of patients. Subsequent low-grade urothelial carcinoma was documented in 9.5% of patients. Progression to high-grade urothelial carcinoma was found in 1.6% patients (1% with muscle invasion). No patients with recurrent PUNLMP or subsequent low-grade carcinoma demonstrated invasion. All patients with PUNLMP exhibiting an inverted growth had no recurrence or progression on follow-up.

**Conclusion:**

In this study, primary PUNLMP recurred primarily either as PUNLMP or low grade urothelial carcinoma. Primary PUNLMP rarely progressed to high grade or invasive carcinoma on long term follow-up. No recurrence or progression was documented on follow-up for PUNLMP that demonstrated exclusively inverted growth.

**Virtual slides:**

The virtual slide(s) for this article can be found here: http://www.diagnosticpathology.diagnomx.eu/vs/1332825572154074

## Background

The inclusion of papillary urothelial neoplasm of low malignant potential (PUNLMP) in the World Health Organization (WHO) 2004 Classification of Tumours of the Urinary System and Male Genital Organs resulted in its wide use in clinical practice[1]. There is however an ongoing debate if it indeed represents a distinct entity which has a very low risk of progression, or if it is indistinguishable from low-grade urothelial carcinoma, and what is the clinical significance and the biologic behaviour of PUNLMP [2-4]. Previous studies have typically addressed this entity in series evaluating the WHO 2004 classification system including other diagnostic categories [5-14]. However, only three studies included more than 100 patients with PUNLMP [5,8,13]. The majority of previous studies were smaller, varied in design, had a patient follow-up of less than 5 years and demonstrated broad range of recurrence and progression rates. Possible confounders, which make comparisons between studies difficult, also included: intra- and inter-observer variability, differences in inclusion criteria, length of follow-up and the definition of progression [15]. The lack of transparent study design is another confounding factor, which further influenced the variations in reported results.

The objective of this study was to examine the recurrence and progression rates after a diagnosis of primary (de novo) PUNLMP in a large regional urology practice, including a group of primary PUNLMP with endophytic growth, which demonstrated exclusive endophytic growth (‘inverted PUNLMP’). Inverted PUNLMP, a recently introduced concept, to our knowledge, has not been previously studied and has uncertain clinical significance and behaviour [16,17]. We deliberately excluded from the study any PUNLMP occurring in association with either previous or concurrent urothelial neoplasm (secondary PUNLMP).

## Methods

### Patient selection

The study was approved by the institutional Ethics Review Board. We identified 196 patients with a diagnosis of primary (de-novo) PUNLMP, without a history of any previously documented or concurrent urothelial neoplasm. These were consecutive cases, diagnosed during a 10-year-period (January 1, 2000 to December 31, 2009) and were diagnosed using consistent criteria, in one institution with a centralized regional urology and uropathology setting. PUNLMP arising in association with a previous or concurrent urothelial neoplasm and consult cases were not included in the study by design. All cases were reviewed by at least two pathologists and 7 cases were excluded (reclassified as low-grade urothelial carcinoma), resulting a final cohort of 189 patients with primary PUNLMP.

### Histopathologic criteria

**Figure 1:**
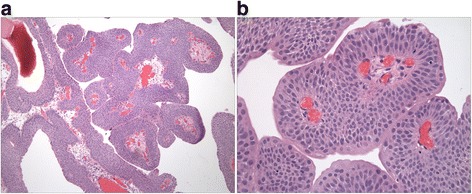
**Papillary urothelial neoplasm of low malignant potential (PUNLMP).** The papillae of PUNLMP are discrete and non-fused **(a)**. They are lined by multilayered urothelium with minimal to absent cytologic atypia and preserved cell polarity **(b)**.

**Figure 2:**
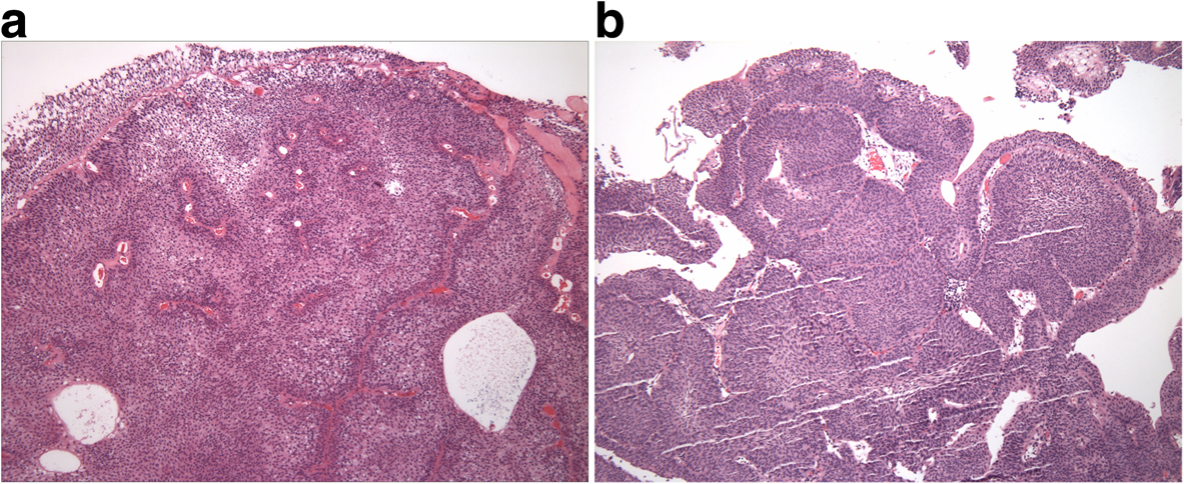
**Inverted papillary urothelial neoplasm of low malignant potential (Inverted PUNLMP).** Inverted PUNLMP demonstrates inverted (endophytic) growth and resembles inverted papilloma, but in contrast, shows expanded and rounded cords and nests, composed of architecturally and cytologically bland urothelial cells **(a and b)**. Inverted PUNLMP also lacks central streaming and peripheral palisading, as typically seen in inverted papilloma.

The PUNLMP diagnosis was based on the WHO 2004 Classification of Tumours of the Urinary System and Male Genital Organs [1]. PUNLMP was defined as a papillary urothelial tumor, which resembles exophytic urothelial papilloma, but shows increased cellular proliferation, exceeding the thickness of the normal urothelium. The papillae of PUNLMP are discrete and non-fused and are lined by multilayered urothelium with minimal to absent cytologic atypia, preserved cell polarity and show only rare mitoses, located basally, as illustrated in Figure 1. Similar neoplasms, which demonstrated inverted (endophytic) growth and resembled inverted papilloma, were also included in the study (labelled ‘inverted PUNLMP’). In contrast to inverted urothelial papilloma, inverted PUNLMP focally showed expanded and rounded cords and nests, which were composed of architecturally and cytologically bland and uniform urothelium, and showed only rare mitoses as illustrated in Figure 2. These neoplasms also lacked central streaming and peripheral palisading, typically seen in inverted papilloma, in the areas with different morphology.

### Follow-up and evaluation of recurrence

Documented recurrence and progression episodes were based solely on the subsequent biopsy findings, typically performed more than 3 months after the initial diagnosis. Recurrence was defined as any subsequent neoplastic lesion, including PUNLMP, low-grade urothelial carcinoma (LGUC) and high-grade urothelial carcinoma (HGUC). Progression was defined as any subsequent higher-grade or invasive lesion, including urothelial carcinoma in-situ.

Follow-up was obtained by review of electronic medical records. The patients with PUNLMP diagnosis typically would have had a follow-up cystoscopy within 3–6 months from the initial diagnosis. The frequency of the subsequent follow-ups and eventual cystoscopies and biopsies would be dependent on the findings at the first follow-up and subsequent clinical symptoms. Descriptive statistical analysis was performed using Microsof Excel software package.

## Results

Of 189 patients with primary PUNLMP, 187 were located in the bladder and 2 were found in the renal pelvis and the ureter (1 each). The data on patient demographics, gender and follow-up are shown in Table 1. Overall, 20.1% (38/189) patients were ≤50 years old and 79.9% (151/189) were > than 50 years old. No cancer-related deaths were documented after primary-PUNLMP in any of the patients.

**Table 1:** **Demographics, gender distribution and followup in patients with primary PUNLMP**

	**All patients with primary PUNLMP**	**All patients with recurrence and progression**	**Recurrence to PUNLMP only**	**Progression to low grade or high grade UC**
Patients, no. (%)	189 (100)	38 (20.1)	17 (9)	21 (11.1)
Age at diagnosis, y median/mean (Range)	66/64 (19–92)	70/66 (44–88)	69/66 (41–83)	71/67 (45–88)
Male: Female	1.7 : 1	2.2 : 1	1.8 : 1	2.5 : 1
Follow up, mo, median/mean (Range)	61/64 (9–128)			

PUNLMP recurrrence and progression data are summarized in Table 2. Overall, 20.1% (38/189) patients demonstrated recurrence or progression with a subsequent urothelial neoplasm, including PUNLMP, LGUC and HGUC (with or without invasion). Recurrent PUNLMP without any grade or stage progression was seen in 9% (17/189) of patients. Grade progression to either LGUC or HGUC was found in 11.1% (21/189) of patients. Grade progression only to LGUC was seen in 9.5% (18/189), while progression to HGUC was found in 1.6% (3/189) of patients). Median time to recurrent PUNLMP was 13 months (mean 20; range 7–78 months), while the time interval to subsequent LGUC was 47 months (mean 42; range 5–87 months). In patients with recurrence or progression, 84% (32/38) were older than 50 years. Overall recurrence or progression was documented in 21.2% (32/151) patients older than 50 years. Of 6 of 38 (15.8%) patients younger than 50 years, 4 had a subsequent diagnosis of non-invasive LGUC and 2 had recurrent PUNLMP; no progression to HGUC or invasive carcinoma was documented in any patient younger than 50. Invasive carcinoma was not seen in any of the 17 patients with recurrent PUNLMP or in any of the 18 patients with subsequent diagnosis of LGUC.

**Table 2:** **Recurrence and progression in patients with primary PUNLMP**

	**Recur to PUNLMP No. (%)**	**Progress to low grade UC No. (%)**	**Progress to high grade UC No. (%)**
Patients with recurrence/progression (N = 38)	17 (45)	18 (47)	3 (8)
Patients with one recurrence	10	12	1
Patients with multiple recurrences (Range)	7 (2 – 6)	6 (2 – 7)	2 (2 – 4)
Median/mean months to recurrence or progression (Range)	13/20 (5 – 78)	47/42 (5 – 87)	31/32 (4 – 61)

In 1.6% (3/189) patients with primary PUNLMP, there was progression to HGUC (all male; age at first diagnosis 69, 72, and 80 years). Two patients with HGUC had invasive carcinoma, while one patient had only non-invasive HGUC. One patient had a recurrent PUNLMP at 12 months after the initial diagnosis, prior to developing muscle invasive HGUC at 61 months. He underwent cystectomy at 65 months, showing no residual invasive HGUC. One patient had multiple biopsies documenting progression to urothelial in-situ-carcinoma (first at 4 months after initial diagnosis) and pT1 invasive HGUC (at 20 months), prior to cystectomy (at 49 months) which showed muscle invasive HGUC with regional positive nodes. One patient progressed to non-invasive papillary HGUC, without any previous recurrences, at 31 months after initial PUNLMP.

### Inverted PUNLMP

Inverted PUNLMP was found in 12 patients, who had a median age of 61 years (mean 58; range, 31 to 74 years) and a male to female ratio of 1:1. None of the 12 primary PUNLMP with inverted growth had a documented recurrence or progression on follow-up (median 24 months; mean 32; range, 2 to 80 months).

## Discussion

With 189 primary PUNLMP patients, the study reported herein, represents the second largest PUNLMP series documented to date. The diagnosis of primary PUNLMP and the recurrences and progressions in this study were predominantly found in individuals older than 50 years, with a male to female ratio of about 2:1. The progressions to HGUC or invasive carcinoma were documented only in male patients, older than 65 years, which suggests that closer follow-up is particularly warranted in these patients, after a primary PUNLMP diagnosis. No patient with primary ‘inverted PUNLMP’demonstrated recurrence or progression on follow-up in this study.

The recurrence and progression rates after PUNLMP reported herein are in the lower end of the previously reported rates, which are quite broad, and range from 16.7% to 62.2% (Table 3). We found a recurrence and progression rate of 20.1%, which is closer to the rates of 17.9% [13], 29.5% [5] and 32.8% [8], documented in the largest previous studies containing more than 100 patients (Table 3). The rate of recurrence to PUNLMP, without any grade or stage progression, was 9% in this study, again, in the lower end of previously reported rates of 3.6% to 51.9% [5,10,12,15,18]. Recurrence with any grade or stage progression was found in 11.1% of patients in this study, with 9.5% progressing to LGUC and 1.6% progressing to HGUC (1% with invasive carcinoma). The previous studies have documented a great variety of grade or stage progression after PUNLMP diagnosis, ranging from 0% to 42%. More specifically, progression to LGUC ranged from 0% to 34%, progression to HGUC ranged 0% to 3.6%, and progression with invasive carcinoma ranged from 0% to 8%.

**Table 3:** **Recurrence and progression after PUNLMP reported in the literature and in this study**

**Study**	**No.**	**Recur (any) (%)**	**Recur PUNLMP (%)**	**Progress (LG, HG, CIS) (%)**	**Progress to LG (%)**	**Progress to HG (%)**	**Progress with invasion (%)**	**Mortality (%)**
[5]	112	29.5	3.6	10.7	3.6	3.6	3.6	2.7
[6]	8	33.3	NA	0	NA	NA	0	0
[21]	20	25	25	0	0	0	0	NA
[7]	68	32.4	NA	0	NA	NA	NA	0
[8]	116	32.8	NA	2.6	NA	NA	NA	0.9
[9]	19	47.4	NA	NA	NA	NA	NA	NA
[22]	29	NA	NA	6.9	NA	NA	6.9	NA
[10]	53	60	30	42	34	0	8	0
[11]	45	62.2	NA	NA	NA	NA	NA	NA
[14]	12	25	NA	8.3	8.3	0	0	NA
[18]	27	51.9	51.9	0	0	0	0	0.0
[12]	85	47.1	47.1	0	0	0	0	NA
[13]	212	17.9	NA	1.9	NA	NA	NA	0
[15]	31	41.9	12.9	29.0	25.8	3.2	0	0
*Current study*	*189*	*20.1*	*9*	*11.1*	*9.5*	*1.6*	*1.0*	*0*
Range	8-212	16.7-62.2	3.6-51.9	0-42	0-34	0-3.6	0-8	0-2.7

The reported variations of recurrence and progression after PUNLMP are likely primarily owing to the inter-observer variations (i.e. the diagnostic criteria used), the different definitions of progression and the differences in the design of previous studies. Inter-observer, and possibly, intra-observer variations, represent a major confounding variable, dependent on the diagnostic criteria used (or their subjective interpretation), and the diagnostic expertise, experience and consistency of the pathologist, which are often related to the type of practice [19,20]. The differences in the definition of recurrence and progression may also be a major source of variation between studies, as noted by Lee et al. [15]. The specific definitions of progression and recurrence with and without grade progression were explicitly stated only in some studies [5,10,14,15,21]. Some studies however considered progression only when there was progression to HGUC [6,11,18] or when the progression resulted in in-situ-carcinoma, invasive carcinoma or metastases [13,22].

Variations in study design further limit the comparisons between the previous PUNLMP cohorts. For example, it was infrequently specified whether only primary (de novo) PUNLMP cases were studied [6,11,18,21] and if secondary and consult cases were evaluated. The method of documenting recurrence or progression also varied significantly among studies. For example, some studies combined cases of recurrence or progression confirmed by biopsy, with cases in which recurrence or progression were identified only on cytology or cystoscopy with fulguration [5,7,11,14,18]. Some studies reported use of adjuvant chemotherapy, in addition to transurethral resection, either in small subsets of PUNLMP patients, or in larger cohorts with urothelial tumors that included PUNLMP patients [8,13,22]. The length of follow-up varied significantly among previous studies as well. Lastly, in some studies, it is unclear if data were derived from a single center or if they were pooled from multiple institutions, potentially introducing greater inter-observer variability and inconsistencies in the follow-up protocols, even within individual studies [7,12,13,18]. In contrast, current study was restricted only to primary PUNLMP cases, and the recurrences and progressions were based solely on tissue biopsy findings, reported in a centralized regional uropathology setting, maintaining a degree of diagnostic consistency and reducing the possibilities that biopsies were misdiagnosed, not accounted for, or performed and read elsewhere. Importantly, no mortality associated with PUNLMP was observed in this study, in keeping with the great majority of previous studies.

In the current practice, PUNLMP and LGUC have an almost identical clinical management [23]. However, LGUC has a 2 to 3 times higher risk of grade/stage progression (range, 4%-18%) [6,8,13,14,18,21,22,24,25] and also carries a higher risk for cancer-specific death (up to 5%) [6,8,13,18,24-26], which is exceptionally rarely documented in PUNLMP. More specific and tailored management of these two entities may emerge based on studies with cleaner and more transparent design, as well as additional molecular and genetic studies, which will hopefully provide additional markers for more precise separation of urologic malignancies. Until these novel clinical markers are fully validated and used in routine practice, the diagnosis and classification of noninvasive urothelial neoplasms will be based strictly on histomorphologic evaluation [13,23].

In this study, we also included 12 PUNLMP cases with inverted growth (‘inverted PUNLMP’). To our knowledge, no previous study has formally acknowledged this architectural pattern, explicitly stated its inclusion in the analysis, or compared its clinical behaviour to the widely recognized, typical exophytic PUNLMP. This is primarily owing to the fact that inverted PUNLMP is a new and recently proposed diagnostic category within the spectrum of urothelial neoplasms with inverted (endophytic) growth, as a counterpart to the typical PUNLMP with exophytic growth [16,17]. Montironi and colleagues in a recent editorial also suggested the term ‘Inverted urothelial neoplasm of low malignant potential (IUNLMP)’, when a papillary component is lacking and only inverted growth exists [27]. Epstein et al. mention the existence of ‘inverted PUNLMP’, stating that it is not currently included in the WHO classification of urothelial tumours and that no explicit definition or nomenclature currently exists for it [16]. In their opinion, if an endophytic urothelial lesion has notably thickened urothelium without architectural and cytologic atypia, the term ‘inverted-PUNLMP’ is appropriate. According to McKenney and Amin, inverted, endophytic PUNLMP patterns exist, but they are rare [17]. Although the group of ‘inverted-PUNLMP’ included in this study is relatively small, no patient with inverted-PUNLMP showed recurrence or progression on follow-up. However, more data are needed to clarify the biologic behaviour and appropriate management of these lesions.

Limitations of the study include its retrospective design and the possible inter-observer variability. We were also unable to accurately determine the prevalence of PUNLMP in our practice, but we would estimate it to be less than 3% of all urothelial neoplasms.

## Conclusions

We found a recurrence rate of 20.1% after primary PUNLMP, in evaluating the long-term outcome of primary-PUNLMP, including PUNLMP with inverted growth (‘inverted PUNLMP’), in a large, single-center study. Recurrence with PUNLMP only was seen in 9% of patients, progression to LGUC only was seen in 9.5% and progression to HGUC was seen in 1.6% of patients. Primary PUNLMP and the subsequent recurrences and progressions were predominantly found in male patients older than 50 years. The progression to HGUC or invasive carcinoma was documented only in male patients, older than 65 years, which suggests that close follow-up is particularly warranted in this patient group, after a diagnosis of primary PUNLMP. The patients with primary inverted PUNLMP had no documented recurrence or progression on follow-up.

## Abbreviations

PUNLMP: Papillary Urothelial Neoplasm of Low Malignant Potential
LGUC: Low Grade Urothelial Carcinoma
HGUC: High Grade Urothelial Carcinoma
WHO: World Health Organization
